# Vascular Remodeling in Pulmonary Arterial Hypertension: The Potential Involvement of Innate and Adaptive Immunity

**DOI:** 10.3389/fmed.2021.806899

**Published:** 2021-12-22

**Authors:** Rachid Tobal, Judith Potjewijd, Vanessa P. M. van Empel, Renee Ysermans, Leon J. Schurgers, Chris P. Reutelingsperger, Jan G. M. C. Damoiseaux, Pieter van Paassen

**Affiliations:** ^1^Division of Nephrology and Clinical and Experimental Immunology, Department of Internal Medicine, Maastricht University Medical Center, Maastricht, Netherlands; ^2^Department of Cardiology, Maastricht University Medical Center, Maastricht, Netherlands; ^3^Department of Biochemistry, Cardiovascular Research Institute Maastricht, Maastricht, Netherlands; ^4^Central Diagnostic Laboratory, Maastricht University Medical Center, Maastricht, Netherlands

**Keywords:** pulmonary arterial hypertension, immunology, vascular remodeling, endotheliopathy, macrophage, histology, immunopathology, interleukin-6

## Abstract

Pulmonary arterial hypertension (PAH) is a severe disease with high morbidity and mortality. Current therapies are mainly focused on vasodilative agents to improve prognosis. However, recent literature has shown the important interaction between immune cells and stromal vascular cells in the pathogenic modifications of the pulmonary vasculature. The immunological pathogenesis of PAH is known as a complex interplay between immune cells and vascular stromal cells, via direct contacts and/or their production of extra-cellular/diffusible factors such as cytokines, chemokines, and growth factors. These include, the B-cell—mast-cell axis, endothelium mediated fibroblast activation and subsequent M2 macrophage polarization, anti-endothelial cell antibodies and the versatile role of IL-6 on vascular cells. This review aims to outline the major pathophysiological changes in vascular cells caused by immunological mechanisms, leading to vascular remodeling, increased pulmonary vascular resistance and eventually PAH. Considering the underlying immunological mechanisms, these mechanisms may be key to halt progression of disease.

## Introduction

**Figure 1:**
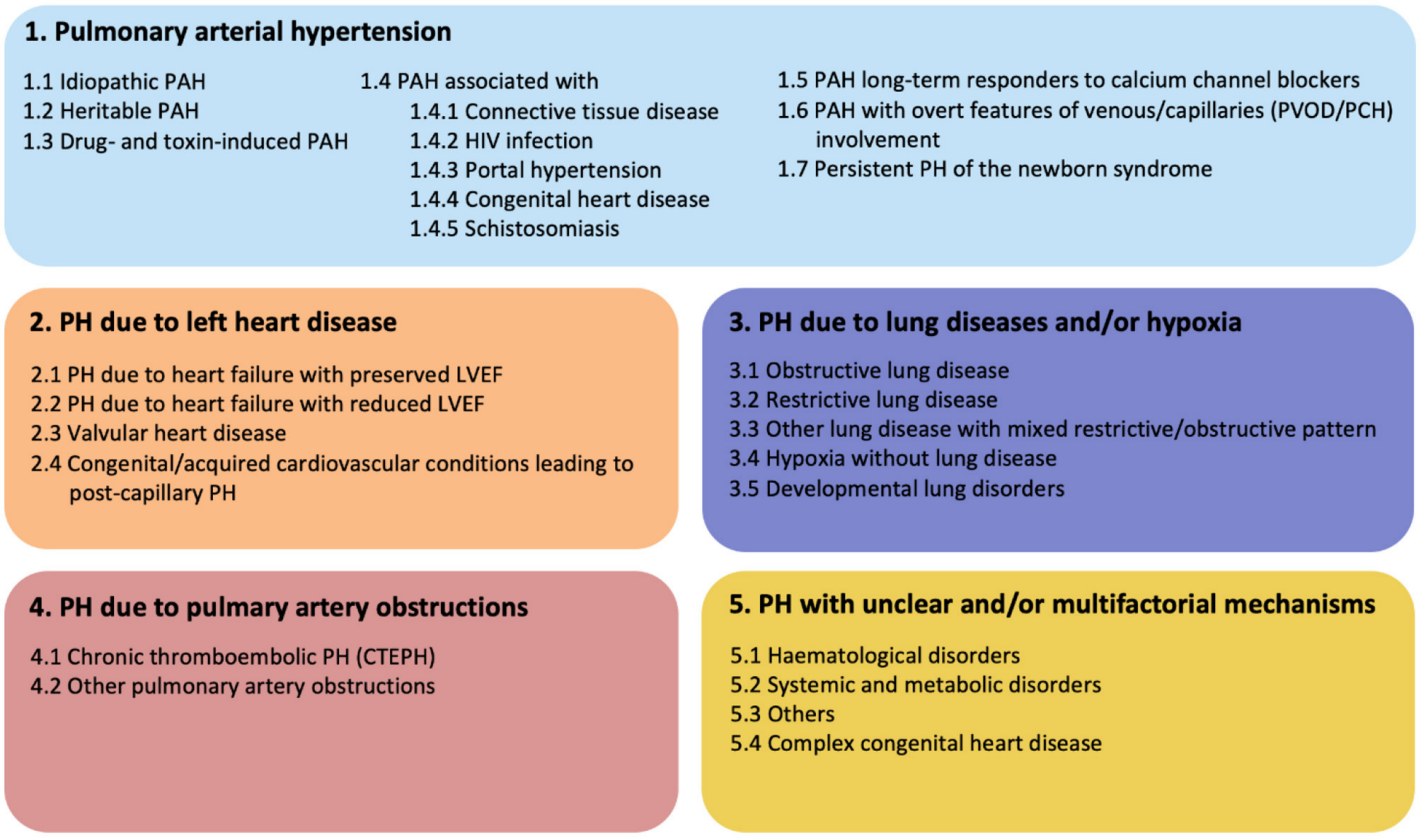
Updated clinical classification of pulmonary hypertension according to the 6th PH world symposium of 2018, Nice, France (1).

Pulmonary arterial hypertension (PAH) is a progressive cardiovascular disease with high mortality and serious impact on the quality of life of affected patients. Due to increased pulmonary vascular resistance (PVR) with pressure overload on the right side of the heart, right sided heart failure and death could be the consequence. According to the recommendation of the 6th Pulmonary Hypertension (PH) world symposium of 2018, PH is diagnosed if mean pulmonary arterial pressure (mPAP) exceeds 20 mmHg, assessed by right heart catheterization (RHC) (1). For many years, the diagnosis of PH was based on mPAP values ≥ 25 mmHg, due to concerns of over-diagnosis and over-treatment. However, the main reason for over-diagnosis appeared the lack of confirmation by RHC. Clinically, it is important to distinguish pre-capillary PAH from post-capillary pulmonary hypertension. This can be determined by measuring the pulmonary arterial wedge pressure (PAWP) and PVR. PAH, also known as pre-capillary PH, is then defined by a mPAP ≥ 20 mmHg and a PAWP ≤ 15 mmHg. The PVR is critical to distinguish post-capillary PH from combined pre- and post-capillary PH. Combined PH is defined as: mPAP ≥ 20 mmHg and PAPW ≥ 15 mmHg, and PVR must be ≥ 3 Wood units. Recognition of these subtypes of PH provides insight into the etiology. The classification of PH according to the most recent consensus meeting is shown in Figure 1 (1). When studying the etiologies of PH, numerous different underlying diseases may contribute to the development of the typical vasculopathy or vascular remodeling that subsequently leads to increased pressure in the pulmonary circulation. Idiopathic PAH (IPAH) is known to feature this pre-capillary vasculopathy, however, without direct contribution of an underlying disease. It is hypothesized that triggers such as infections or toxins inducing endothelial cell injury and activation play an important causative role. PH could also be caused by left sided heart disease, chronic lung disease, chronic thrombo-embolic events (CTEPH) or caused by multifactorial triggers (Figure 1). Interestingly, PH is often associated with systemic autoimmune diseases and the role of the immune system in PH has been studied for decades. Examples of autoimmune diseases with increased risk of developing PAH are in particular systemic sclerosis (SSc) and mixed connective tissue disease (MCTD). However, PAH may also occur in a minority of systemic lupus erythematosus (SLE) and anti-phospholipid syndrome (APS) patients, the latter in the context of CTEPH (2). Involvement of the vasculature, inflammation and stronger clotting tendency, is core to the pathologic process in these conditions. However, the underlying mechanisms are very different. Remarkably, ANCA-associated vasculitis, a systemic disease that predominantly affects small and medium-sized vessels, is almost never complicated by PAH. These observations highlight the complexity of PAH and its association with very specific pathological mechanisms leading to vascular remodeling seen in PAH. Other factors also may contribute to the pulmonary risk in these systemic autoimmune patients. SLE and anti-phospholipid syndrome APS are associated with hyper coagulability, and can cause chronic thrombo-emboli and therefore may develop CTEPH (3). SSc can induce pulmonary fibrosis leading to hypoxic vasoconstriction with long term vascular remodeling. Patients with SSc are at risk to develop cardiac fibrosis or even pulmonary venous occlusive disease (PVOD), leading to PH in a pathophysiological different manner (4). In the PH classification (Figure 1) these patients are clustered as connective tissue disease associated PAH (CTD-aPAH). This cohort also includes numerous patients with the rare presentation of PAH and coexistent rheumatoid arthritis, giant cell arteritis, and myositis, either or not in combination with interstitial lung disease. Altogether, we must better understand how precapillary PAH develops, and thereto need to study the pathways of immune-activation, how autoimmunity develops, and how and by which effector mechanisms the early signals translate into long term downstream pulmonary vascular changes. Vasculopathy in PAH has been observed in all vessels of the pulmonary arterial circulation. Plexiform lesions are typical for PAH, and have a possible role in shunting between pulmonary and bronchial circulation. Other lesions, like intimal thickening, adventitial fibrosis and arterialization of non-muscular vessels, are also observed in PAH and contribute to increased PVR. Many studies have reported insights in the process of vascular remodeling in PAH, indicating that both innate and adaptive immune system are strongly involved. However, it is still not fully understood how cells of the immune system induce changes to the pulmonary vascular wall and how this could lead to PAH. The scope of this review is focused on the immunological mechanisms that contribute to vascular remodeling in PAH with a particular focus on idiopathic and CTD-aPAH. Additionally, we focus on the interaction between vascular stromal cells and immune cells. Finally, possibilities for future research and clinical implications will extensively be discussed.

## Histopathology of Vascular Remodeling in Pulmonary Arterial Hypertension

**Figure 2:**
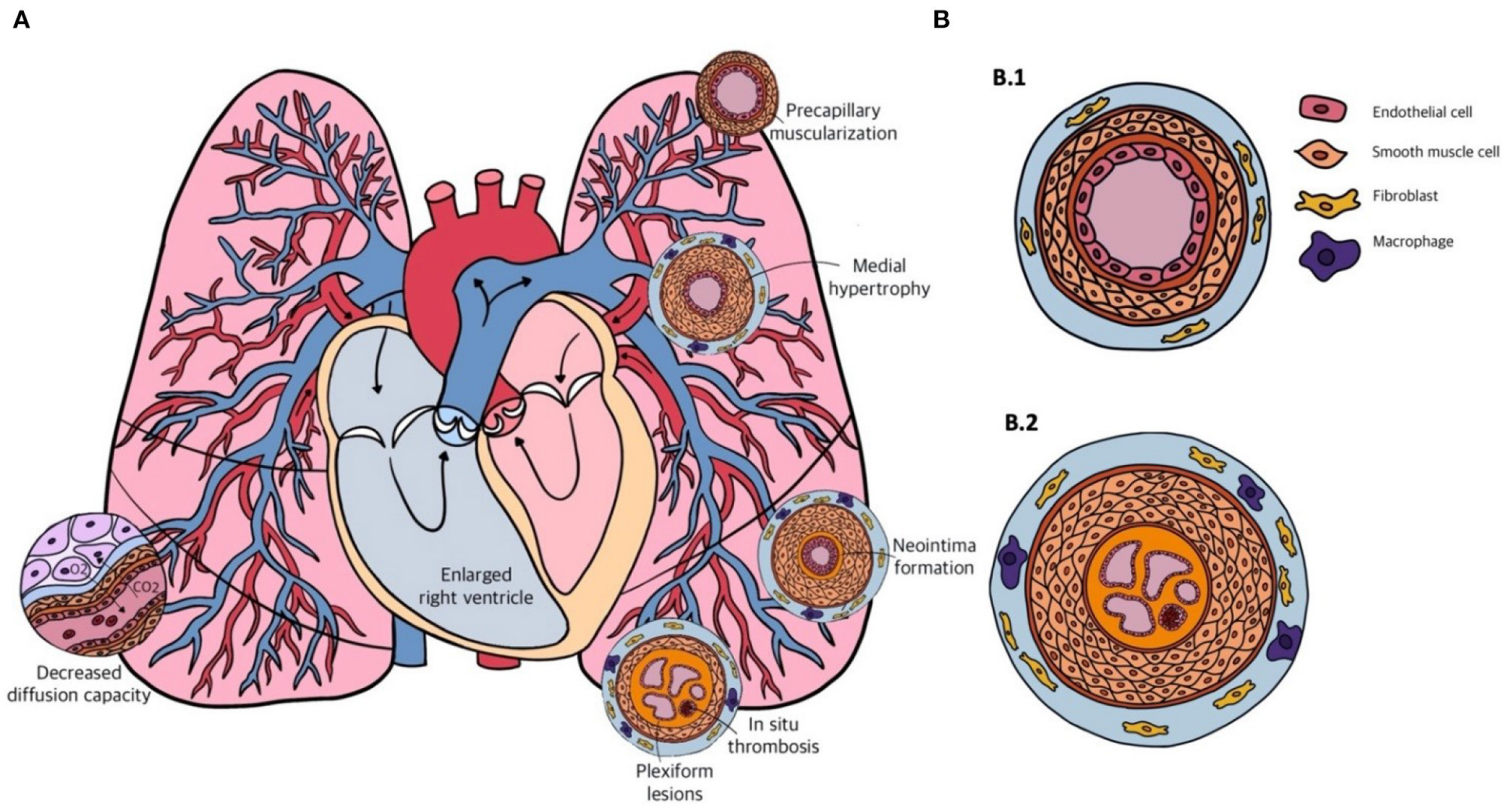
**(A)** Schematic overview of the cardiopulmonary system in pulmonary arterial hypertension (PAH) and the different forms of vascular remodeling. **(B)** Transversal sections of the pulmonary arterial vasculature in a healthy person (B.1) and in a patient with end-stage PAH (B.2). Showing from inside to outside: multiple capillary channels typical for plexiform lesions, with *in-situ* thrombosis, intimal thickening, pulmonary artery smooth muscle cell (PASMC) proliferation in the medial layer, and infiltration of fibroblasts and macrophages in the adventitial layer.

PAH is considered to be a pan-vasculopathic disease of the pre-capillary compartment of the pulmonary circulation. Nevertheless, histology shows that different phenotypes of vascular remodeling in PAH occur depending on the size and function of the different vessels. These vessels are the distal muscular arteries with a diameter of 70–500 μm, small pre-capillary pulmonary arterioles ranging from 20 to 70 μm, and small capillaries that have diameters smaller than 20 μm. Lesions that are observed in the distal muscular arteries consist of medial hypertrophy and hyperplasia, intimal and adventitial fibrosis and *in situ* thrombosis or plexiform lesions (Figure 2). Small arterioles and capillaries often show obliteration, muscularization and perivascular inflammation. Taking the different layers of the vascular wall as a starting point, we will elaborate on the possible mechanisms that underlie the different lesions that are seen in PAH.

### Intimal Remodeling, Plexiform Lesions and Pericytes

The intimal layer primarily consists of an endothelial monolayer. In severe PAH, with mPAP pressures higher than 45–50 mmHg, the intimal fractional thickness is increased up to three fold as is observed in some 25% of the patients with PAH (5). This results in an increase of pulmonary vascular resistance (PVR) by 40 times. The thickened intimal layer consists predominantly of collagen and mucin rich matrix, fibroblast-like cells, endothelial cells, as well as pulmonary arterial smooth muscle cells (PASMCs). The common denominator for the development of irreversible vascular remodeling in PAH is an altered crosstalk between cells in the vascular wall, this concerns particularly the endothelial cells lining the intimal layer. The endothelial cell is known as a critical source of key mediators for vascular remodeling like growth factors [fibroblast growth factor (FGF)-2], serotonin (5-HT), angiotensin II (AngII), vasoactive peptides like nitric oxide (NO), prostaglandin I2 (PGI2), endothelin-1 (ET-1), cytokines like interleukin-1 (IL-1), IL-6 and chemokines (6–9). Overproduction of these paracrine mediators has a direct effect on the proliferation of other cells in the vascular wall, like PASMC, pericytes or endothelial cells themselves (autocrine effects), contributing to intimal remodeling (10, 11).

When endothelial cells proliferate in an overshooting regenerative manner, they can form plexiform lesions. These lesions are the classic histological hallmark of PAH, and are mainly seen in severe or progressive PAH (12, 13). Plexiform lesions are often located at vascular branching points and contain vascular channels, that are highly ordered. The vascular channels in plexiform lesions are lined with intact endothelium, that is separated by intermediate PASMCs with in-between synthetic and contractile phenotypes. Both phenotypes are necessary for progressive vascular remodeling. Plexiform lesions often resemble glomeruloid-like lesions with sprouting of new blood vessels, and excessive expression of angiogenic markers like vascular endothelial growth factor (VEGF), and hypoxic inducible factor-1α (HIF-1α). This suggests a process of disordered angiogenesis (14). Plexiform lesions are more often seen in IPAH, but can also be found in some 50% of the CTD-aPAH cases, with similarities in composition, architecture and microenvironment (5). Inflammatory cells in these lesions are a mixture of T-cells (CD3^+^), monocytes and macrophages (CD68^+^) and tryptase positive mast-cells in both IPAH and CTD-aPAH (15). Later in this review, the role of these cells in PAH will be further discussed.

Plexiform lesions are typical features of long standing vascular remodeling in PAH. Moreover, they may also have functional implications in vascular remodeling. Based on findings of close association of plexiform lesions and dilated bronchial microvessels in patients who died due to severe IPAH, plexiform lesions are suggested to function as anostomotic structures between the pulmonary and bronchial circulation (16). Hemodynamic stress, caused by these anastomoses, could lead to vascular wall stretch in the bronchial circulation and expansion of the vasa vasorum of pulmonary arteries. This could provide a pathway for progenitor and inflammatory cells to participate in pulmonary arterial remodeling. Nevertheless, Ghigna et al. showed that bronchial artery hypertrophy and bronchopulmonary shunting was also associated with post-capillary remodeling (17). This occurred more frequently in patients with genetic BMPR2 mutations. They also described the newly found singular millimetric fibrovascular lesions (SiMFis), which were also associated with these genetic BMPR2 mutations. A direct relationship between the degree of bronchial vascular remodeling and disease severity, based on PVR, mPAP or cardiac index, was not observed in this study.

Pericytes are important supporting cells that maintain endothelial viability in angiogenesis, a process in which new vessels sprout from existing vessels. Recently, the role of pericytes in the systemic vascular changes of diabetic retinopathy and stroke has been investigated (18–22). In these cases, the loss of pericytes caused vascular dysfunction and loss of vessels. However, the role of pericytes in the pulmonary vasculature is less clear. Ricard et al. showed that increased pericyte coverage of the distal arterial vasculature in PAH correlated well with vascular remodeling and that this process was stimulated by FGF-2 and IL-6 (11). TGF-β in this study affected the capacity of pulmonary pericytes to differentiate into smooth muscle-like cells, thereby contributing to increased PVR and PAH development. Dierick et al. studied the role of pulmonary resident PW1^+^ progenitor cells in hypoxia-induced PH (23). They showed that hypoxia-induced vascular muscularization is dependent on PW1^+^ cells, and showed that some of these cells also express pericyte markers. Most likely mediated by stimulation by CXCR-4. This is mainly the case in neo-muscularization of non-muscularized vessels in PH. Nevertheless, Crnkovic et al. studied the involvement of other cell lineages that contribute to SMC differentiation and showed that resident SMCs are the major source of α-smooth muscle cell actin^+^ cells that appear in the remodeled pulmonary arteries (24). Besides, endothelial cells were also covered in the lineage tracing experiments of Crnkovic et al., but due to the complexity and limitations of the current murine models, a definitive conclusion on the subject of endothelial mesenchymal transition (endoMT) remains absent. However, endothelial cells are substantially involved in the biology of SMCs in vascular remodeling in PAH. Sheikh et al. showed that enhanced HIF1-α upregulates endothelial cell secretion of platelet derived growth factor-B (PDGF-B), which induces priming of SMCs, making them more susceptible to induce distal arteriolar muscularization (25). Bordenave et al. also investigated the role of pericytes in distal arteriolar muscularization (26). They confirmed that pericytes are mobilized to muscularized distal arterioles in PAH and demonstrated that pulmonary pericytes are altered in patients with IPAH, overexpressing CXCR-7 and TGF-βRII. Although also other cell lineages may contribute to neo-muscularization and vascular remodeling in PAH, pulmonary pericytes orchestrate multiple critical functions that can directly and indirectly contribute to this process. Yuan et al. showed that the disturbed interaction between endothelial cells and pericytes leads to vascular remodeling and is mediated by pyruvate dehydrogenase kinase 4 (PDK4), which is a key player in mitochondrial metabolism and can therefore influence down-stream effects of glycolysis (27). Targeting these pathways may resolve pericyte and endothelial cell interactions and contribute to rescuing vascular remodeling.

### Medial Remodeling

The medial vascular wall is also known to play a major role in vascular remodeling in PAH and primarily consists of PASMCs (28). Stacher et al. have shown that in PAH the media fractional thickness may increase to 20%, and in combination with the increased intimal thickness this correlates well with the increase in mPAP and PVR (5). PASMC proliferation in PAH is a key feature of vascular remodeling and has been thoroughly investigated. Already in the early 80s, animal studies using either hypoxia or monocrotaline (MCT) gave insight into the course and specific anatomical location of PASMC proliferation. In a hypoxic rat model, the onset of proliferation was observed in the hilar vessels, predominantly in the intimal and adventitial layer, but with minimal changes in the medial layer (29). In the MCT rat model similar observations were made (30). However, an important finding was the decrease of PASMC proliferation in the hypoxic models after 4–5 weeks, suggesting that active PASMC proliferation is a sign of early disease (31–33). This has also been observed in a pathologic study of human tissue of IPAH and hereditary PAH patients (HPAH), in which there was no active PASMC proliferation in end stage lung tissue (34). As mentioned before, endothelial cells contribute significantly to PASMC proliferation (10). However, many of the paracrine mediators remain still unknown. On top of the PASMC proliferation in vessels with an already existing medial layer, vessels without a distinct medial layer become muscularized (35). This is often accompanied by neointima formation. The distal extension of smooth muscle arteries into small peripheral, normally non-muscular, pulmonary arteries is commonly seen in vascular remodeling in PAH, leading to increased vascular tone and PVR. A new perspective on PASMCs in vascular remodeling in PAH is cellular senescence (36). Cellular senescence is defined as irreversible loss of cell growth and proliferation, which is mainly characterized by the cessation of cell replication. PASMC senescence in PAH is induced by hypoxia and plays a key role in hypoxia-induced PASMC proliferation (36). A fraction of the PASMCs become senescent which promotes paracrine IL-6 release, that is mediated by the mTOR/S6K1 pathway which accelerates PASMC senescence, and showing the important role of immune mediated mechanisms in vascular remodeling in PAH.

### The Adventitial Layer as an Inflammatory Signaling Hub

The adventitial layer of the vessel wall is a fascinating compartment but its role in vascular remodeling is debated and somewhat controversial. The “inside-out” hypothesis of vascular remodeling, as was mentioned earlier, states that endothelial dysfunction, either caused by inciting factors coming from the blood, or by intrinsic endothelial cell abnormalities, results in intima fibrosis, PASMC proliferation and migration and the formation of neointima. However, the “outside-in” hypothesis recently claimed a more dominant role for the adventitial layer in vascular remodeling. Adventitial thickening in PAH is mild, or lacking, as shown in histopathological studies by Stacher et al. (5). In contrast, Chazova et al. reported two- to four-fold increase in adventitial thickness in an autopsy series of 19 IPAH patients compared to controls (28). The adventitial layer is physiologically complex and consists of numerous cell types such as fibroblasts, immune modulatory cells, resident progenitor cells, endothelial cells of the vaso-vasorum and even adrenergic nerves (37). In comparison, intimal and medial “monocellular” layers consist of endothelial cells, pericytes and PASMCs only. Although structural changes in the adventitia have less direct impact on PVR than thickening of intima and media, it plays a major role as inflammatory signaling hub and facilitates feed-forward interactions between incoming macrophages and resident fibroblasts (38, 39). The “outside-in” hypothesis will be extensively discussed in the next section.

### The Histopathology of Vascular Remodeling in Summary

The aforementioned studies, all show some involvement of immune cells through the different layers of the vascular wall in vascular remodeling in PAH, and suggest a dynamic interaction with stromal vascular cells. The thickness of the vessel layers correlates well with increased PVR and thereby development of PAH, with maybe the exception of the adventitial layer. However, fractional thickness is not the only cause of PAH development. How the state and timing of activity of different immune cell types relate to vessel wall changes and increased PVR, is not fully clear from available studies. This will be of interest particularly to better understand (peri)-vascular inflammation and the interactive role between the vascular wall and immune system and how this leads to extensive vascular remodeling in development of PAH. This may also lead to novel targets of therapy for PAH.

## Perivascular Inflammation and The Interplay Between Immune Cells and Vascular Stromal Cells in Vascular Remodeling in PAH

Vascular infiltration of immune cells, consisting of both innate and adaptive origin, in PAH has been observed in many different studies. Marsh et al., investigated lungs of PAH patients with end-stage disease by using flow cytometry (40). They observed increased activated plasmacytoid dendritic cells (pDCs), macrophages, mast cells and T-cell receptor γδ (TCR-γδ) T-cells, which suggests a link between innate and adaptive immunity. TCR-γδ T-cells are important in tissue homeostasis and wound healing by releasing insulin-like growth factor-1. This enhances PASMC proliferation and, as mentioned before, dysregulated proliferative PASMCs are a hallmark of vascular remodeling in PAH (41). pDCs produce substantial amounts of type-1 interferon, which has been implicated in PAH pathogenesis as well. Type-1 interferon can also induce anti-inflammatory effects by activating Tregs (42, 43). Due to lack of cell subtype specific markers in the Marsh study, it is unclear what type of macrophages infiltrated into the vessel wall. However, this study extracted cells from whole lung tissue samples, thereby not only focusing on immune cells in pulmonary vessels, but also in parenchymal pulmonary tissue as well. Nevertheless, this may immunologically be as important. Perros et al. have shown the presence of pulmonary lymphoid neogenesis and composition of tertiary lymphoid tissue in IPAH (44). Colvin et al. have shown that in PAH, bronchus-associated lymphoid tissue expands and contributes to auto-immunity and auto-antibody development (45).

Although we would like to draft a clear overview of the main functions of each immunological cell in vascular remodeling in PAH, it is unfortunately not preferable. Due to the dynamic nature and complexity of both the immune system and vascular remodeling itself, we preferred to focus on the dynamic interplay between both the immune cells and vascular cells mediated by many different cytokines and chemokines altogether. However, elucidating the mechanisms in this way may be more challenging. Nevertheless, by discussing these mechanisms in this way we preserve a more natural depiction of the patho-immunological mechanisms involved in vascular remodeling in PAH. Due to the both advancing clinical implications of interleukin-6 (IL-6) and it being the most discussed immunological mediator in PAH, we decided to discuss the role of this cytokine in a separate paragraph.

### Fibroblasts and M2 Polarized Macrophages

El Kasmi et al. showed promising results regarding effects on vascular remodeling by cells in the adventitial layer of the vascular wall in PAH (46). This study showed an increase in CD68^+^ cells (monocytes and macrophages) in the adventitial vascular layer in human IPAH, hypoxia induced PH in calves and MCT rats. These CD68^+^ cells expressed CD163 and CD206, known to be M2-like macrophage subtypes. Interestingly, Signal Transducer And Activator Of Transcription 3 (STAT3) expression was upregulated in these cells, which suggests a different polarization mechanism than the conventional IL-4/IL-13 mediated pathway. The latter is usually induced via STAT6 expression. Their findings indicate that M2 polarization can also be mediated by IL-6. Kasmi et al. also showed that conditioned media of adventitial fibroblasts from different PH models, and from humans with PAH, in contact with macrophages induce a shift to the M2 subtype. Min Li et al. investigated the distinct phenotypical changes of these adventitial fibroblast after exposure to chronic hypoxia (47). This phenotype, that is also known as a pulmonary hypertension fibroblast (PHFib), was characterized by high expression levels of canonical pro-inflammatory cytokines (IL-1β, IL-6), macrophage chemo-attractant cytokines CCL2 (MCP-1), CXCL12 (SDF-1), CCL5 (RANTES), macrophage growth factor (GM-CSF), CD40L and VCAM-1, contributing to M2 macrophage subtype polarization. This study also showed that PASMCs that were harvested from the same vessels, do not show a different phenotype. However, fibroblasts seem to differentiate into pro-fibrogenic or PASMC-like subtypes, which suggests a broad involvement in vascular remodeling in PAH.

The role of other chemokines in PAH has also been studied. Amsellem et al. investigated the CX3CL1/CX3CR1 and CCL2/CCR2 (better known as MCP-1) chemokine systems in hypoxia induced PH (48). The impact on monocyte trafficking, macrophage polarization, and pulmonary vascular remodeling was addressed. CX3CR1^−/−^ mice were protected against hypoxic PH compared to wild-type mice, whereas CCL2^−/−^ mice and double CX3CR1^−/−^/CCL2^−/−^ mice exhibited similar PH severity as did wild-type mice. CX3CR1 deficiency showed an increase in both the number of lung monocytes as well as macrophage perivascular inflammation and most importantly, a shift from M2 to M1 macrophage polarization. The absence of M2 macrophages, therefore, seems to prevent development of PH. CX3CR1 deficient mice showed diminished PASMC proliferation, which was partly mediated by CX3CL1 secretion. This study suggests a role of M2 macrophages as effector cells in PH. Supernatant from hypoxia induced-M2 macrophages induces PASMC proliferation, and is also able to induce endothelial cell injury, mediated by leukotriene B4 (LTB-4), contributing to the outside-in hypothesis (49, 50). M2 macrophages also exhibit functions on the vascular wall that do not involve vascular stromal cells, for instance excreting extracellular matrix (ECM). Legumain is a newly discovered cysteine proteinase belonging to the C13 peptidase family and is primarily expressed in macrophages. Legumain increased the synthesis of extracellular matrix (ECM) proteins via matrix metalloproteinase-2 (MMP-2) activation, promoting vascular remodeling in PAH (51).

The study of Hashimoto-Kataoka et al. showed the dynamic interplay between cytokines, adaptive and innate immune cells and eventually PASMC proliferation (52). Firstly, they showed that IL-6 blockade ameliorated hypoxic pulmonary hypertension (HPH) in mice, which also prevented the hypoxia induced accumulation of Th17 cells and M2 macrophages in lungs. IL-17 blockade had no effects on HPH, though IL-21 knockout mice were resistant to HPH. These knockout mice also didn't accumulate M2 macrophages in their lungs, once again suggesting an important role for M2 macrophage in PH. IL-21 induced M2 macrophages were cultured and PASMCs were then incubated with conditioned medium of these macrophages. Interestingly this induced significant PASMC proliferation. Investigating the molecular mechanism behind proliferation, it was shown to be related to CXCL12, that normally acts as a chemokine that stimulates PASMC proliferation by binding to CXCR4. When CXCR4 was antagonized, PASMC proliferation did not occur after incubation of PASMCs with the conditioned media of the M2 macrophages. Hypoxia did also increase the number of Ki67 positive PASMCs, indicating increased proliferation. IL-21 knockout mice had significantly less Ki67 positive PASMCs. To validate the clinical significance, IL-21, Arg-1 and CD206 cells (M2 macrophage subtype markers) were stained in tissue of IPAH patients undergoing lung transplantation and controls. This data showed significant increase of both IL-21 producing cells, and M2 macrophages in the adventitial layer. Concluding, M2 macrophages play a major role as effector cells in vascular remodeling in PAH. M2 macrophages are known for their heterogeneity, and future studies should focus on specific M2 macrophage subtypes and their specific interactions with vascular stromal cells in PAH.

### Mast-Cell—B-Cell Axis and Humoral Immunity in PAH

It is known that tissue resident mast cells, B- and T-cells are increased in patients with diverse forms of PAH. These different cell types have an important direct effect on the vasculature, especially by their involvement on the immunological micro-environment by secreting chemokines and cytokines that promote further vascular low-grade inflammation.

Breitling et al. studied the interaction between mast-cells and B-cells mediated by IL-6 in PH rat models (53). This study revealed an important link between mast cells and B-cells with an interplay of IL-6 in the MCT and aortic banding rat models. They showed that mast-cells in PH produce substantial amounts of IL-6 which promotes B-cells to differentiate into plasma cells, which is in line with the observed up-regulation of immunoglobulin production (53). Aortic banding, showed a drastic increase in total IgG over a period of 9 weeks, and when IgG was purified and transferred to healthy animals, PH developed, suggesting a pathognomonic mechanism of auto-antibodies in PH. Upregulation of IL-6 also showed abundance of T-cells in an IL-6 dependent and mast-cell dependent manner, which suggests a broader role of IL-6 than the mast-cell—B-cell axis only (54). When rats were treated with mast-cell stabilizer ketotifen, IL-6 levels virtually returned to zero.

Different auto-antibodies against endothelial cells (55, 56), phospholipids (57), fibroblasts (58), and nuclear antigens have been described in PAH. Tamby et al. observed the presence of anti-endothelial cell antibodies (AECAs) in IPAH, which hinted at a possible role of the humoral immune system in the pathogenesis of PAH (55). However, not all PAH patients display circulating AECAs (56). AECA is an umbrella term for antibodies against many unknown endothelial antigens. Some groups have tried to identify AECA antigens, but it is still not known to what extent distinct AECAs may exhibit clinical significance. The group of Dib et al. identified targets in patients with SSc, with or without PAH, and in patients with IPAH (59). They observed AECA binding to lamin A/C and the tubulin β-chain, both intracellular proteins. However, hypothetically membrane surface antigens are the main antigens for AECA binding *in vivo*. It is still unknown if and at what stage of disease AECAs may play an important role. Our group has investigated the role of pathogenicity of AECAs (60). We showed that the presence of AECAs contributed to increased vascular inflammation by increased production of pro-inflammatory cytokines, like IL-6, chemokines IL-8 and CCL2 (MCP-1) and an increased expression of adhesion molecules like intercellular adhesion molecule 1 (ICAM-1) and VCAM-1. These molecules are known to promote vascular inflammation and vasculopathy, and could therefore contribute to vascular remodeling in PAH. However, when isolated human umbilical vein endothelial cells (HUVECs) are exposed to the AECAs of IPAH and SSc patients, there is no interplay with other immune cells in this model. The dynamic process of inflammation cannot be tested on isolated cell cultures. Therefore, it is of great importance that other *in vitro* models, besides already existing animal models, will be utilized. Tissue engineered vessel-on-a-chip, or so-called organoids to assess vascular remodeling by using different co-cultures of vascular stromal cells in contact with patient immune cells, sera or isolated immunoglobulins could be a viable option. Patient specific antibody—antigen matching by differentiating patient-derived pluripotent stem cells (iPSCs) into endothelial cells could help discovering more relevant antigens that may play a role in the immune mediated vascular remodeling in PAH.

### Complement Mediated Vascular Remodeling

The complement system is a very complex and evolutionary old immunological protein cascade with 3 different pathways known as the mannose binding lectin, classical and alternative route. The complement system has multiple effector mechanisms e.g., chemotactic, opsonic, pro-inflammatory and membrane attack complex-mediated lytic mechanisms. The role of the complement system in PAH has not been investigated to great extent, especially in the case of local inflammation of the tissues. However, Stenmark and colleagues recently studied the role of the complement system in vascular remodeling in PH. They particularly looked at the role of complement in the early phase of disease in rat and mouse hypoxic models. Lungs of these mice and rats demonstrated C3 depositions both peri-bronchial as well as perivascular, with high levels of anaphylatoxin receptor expressing cells. Complement depositions were related to localized proliferating cells as measured by Ki67, and increased secretion of GM-CSF and MCP-1 stimulating macrophage inflammation. RNA sequencing suggested hypoxia induced alternative complement pathway activation, mediated by its activator complement factor b (Cfb). Analysis of serum complement markers, suggested the alternative complement cascade as a biomarker in PAH. Hypoxia also induced luminal and medial IgM depositions, whereas IgG deposits were found more perivascular. Immunoglobulin knockout models showed lack of complement activation, reduced perivascular accumulation of CD68^+^ macrophages and decrease in both Ki67 positive cells and chemoattractant secretion. After immunoglobulin restitution, these effects were reversed, proposing that complement mediated perivascular lung inflammation in PAH is immunoglobulin mediated and, as such, involves the classical pathway. Other more chronic PAH hypoxia animal models and lung tissue of IPAH patients showed C3d (complement 3 degradation) depositions suggesting the longitudinal involvement of complement in PAH. Functional analyses of the complement system in a clinical setting, however, may be informative in pointing toward underlying antibody-mediated diseases such as SLE or active cryoglobulinemia.

### The Role of IL-6 in Vascular Remodeling in PAH

Clinically, IL-6 serum levels in severe PAH have already been studied for over 25 years. Humbert et al. showed increased serum levels of IL-6 compared to controls and chronic obstructive pulmonary disease associated PAH (COPD-aPAH) patients, suggesting that IL-6 may be an important mediator in PAH (61). Since then, many other groups have confirmed this observation in mild PAH as well. However, most of these studies were conducted in small single center cohorts composed of primarily severe IPAH patients, making it unclear if this increase in IL-6 serum levels was due to critical illness or if it was a driving factor in vascular remodeling. Simpson et al. addressed this important issue, and investigated the association between IL-6 and its cellular sources in different clinical phenotypes of PAH (62). This study showed that IL-6 was produced by vascular cells, where after they investigated the role of IL-6 as a mechanistic biomarker in early disease in different subtypes of PAH. Simpson et al. showed that PASMCs are one of the major vascular cells that produce IL-6, besides the perivascular infiltrates of inflammatory cells. This pathogenic role of IL-6 in PAH has been confirmed by others.

Steiner et al. showed that over-expression of IL-6 in transgenic mice under normoxic conditions leads to development of elevated right ventricular systolic pressure as well as vasculopathic changes seen in PAH (63). This effect was even larger under hypoxic circumstances. Vascular homeostasis is highly dependent on the balance between apoptosis and proliferation of the individual vessel wall cells. Up and down regulation of factors that maintain vascular homeostasis based on a correct balance between apoptosis and proliferation were dysregulated in these transgenic IL-6 mice. This suggests that IL-6 may induce development and progression of vascular remodeling in PH, independently of hypoxia, via pro-proliferative anti-apoptotic mechanisms. This was also seen in the new MRL/lpr autoimmune mouse model for PH, which develops hypergammaglobulinemia, produces various auto-antibodies (for instance anti-dsDNA), and spontaneously develops vasculitis and nephritis (64). Although, the pulmonary consequences were never well studied until now, this mouse model seems to induce PH with SLE-like characteristics. Interestingly, this occurred by inducing disturbed vasomotility via increased prepro-ET1 (prepro-endothelin 1) and decreased eNOS (endothelial nitric oxide synthase) activity. IL-6 was also upregulated and led to vascular remodeling with increased PASMC proliferation and decreased apoptosis, and eventually PAH. The latter is in concordance with other studies, like the study of Le Hiress et al. in which specific cytokines, including IL-6 and upregulation of ICAM, VCAM and E-cadherin were observed due to dysfunctional endothelial cells, without presence of a distinct immunological disease (65). Our clinical experience with immunology in PAH patients for over 15 years showed us that many PAH patients show signs of immune mediated disease, without confirmation to a specific auto-immune disease or clinical diagnosis. Biomarkers like soluble IL-2 receptor and IL-6 may be elevated, or auto-antibodies are present and may contribute to the development of PAH, without developing full symptoms as seen in connective tissue diseases. Immune activation or dysregulation may lead to increased IL-6 levels in pulmonary tissue and vessels and may, just as in animal models, contribute to development of PAH most likely in a multi-hit manner. Savale et al. showed that knock-out of IL-6 in mice, attenuates PH development induced by hypoxia (66). *In vitro* studies showed increased IL-6 secretion by PASMCs with a further increase after hypoxia. IL-6 knock-outs showed less perivascular infiltration of immune cells and less pronounced arterial muscularization, suggesting a role for IL-6 in modulating lung vessel inflammation and remodeling during hypoxic PH progression as well. Tamura et al. showed insights into the direct underlying mechanism of IL-6 on the vascular wall. They elucidated the role of ectopic membrane-bound IL-6 receptor upregulation in PASMCs in PH and showed that IL-6 induces overexpression of anti-apoptotic proteins like MCL-1 and BCL2. This led to excessive accumulation of PASMCs within the pulmonary arterial vasculature (67). They also showed that deletion of the IL-6 receptor or blocking via anti-IL-6 receptor antibodies (tocilizumab), leads to resistance to experimental PH development under hypoxic circumstances.

### Inflammatory and Immunological Mechanisms in Summary

**Figure 3:**
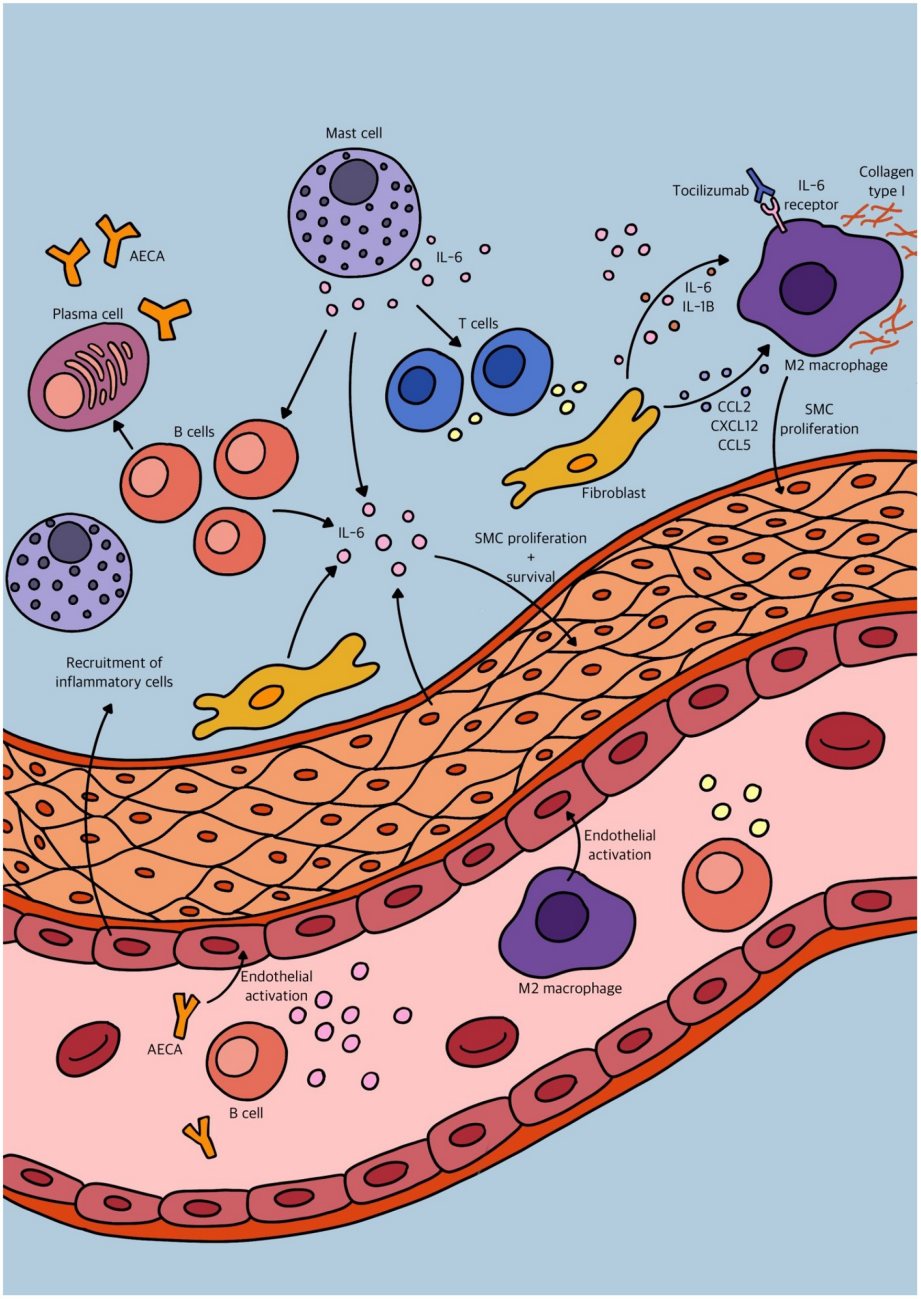
Schematic overview of some of the different immunological mechanisms involved in vascular remodeling in pulmonary arterial hypertension (PAH).

Traditionally, vascular inflammation has been considered an “inside-out” response centered on neutrophil granulocyte, monocyte and macrophage recruitment from the lumen to the intima of blood vessels after endothelial cell injury or activation. However, growing evidence supports a new paradigm of an “outside-in” hypothesis, in which vascular inflammation is initiated and/or perpetuated by adventitial fibroblasts. The aforementioned studies substantiate this “outside-in” hypothesis, that in combination with other findings may be triggered by changes of the intimal vascular layer. How these mechanisms develop over time and what the underlying trigger may be, like antibodies targeting cells of the vascular wall, should be investigated in an immunological approach. We tried to depict an overview of the many different immunological mechanisms involved in vascular remodeling in PAH in Figure 3. However, histological proof of active inflammation in vascular remodeling in PAH patients remains limited. This might be due to a relative contra-indication of video assisted thoracic surgery (VATS) procedures in PAH patients (68). Therefore, histological analysis is mostly performed on post-transplant and post-mortem material. However, a quenched representation of immune cell involvement due to end stage disease must be considered. The interaction of both stromal vascular cells and immune cells is dynamic and complex but can be studied in *in vitro* models like organoids, optionally made viable by inducible pluripotent stem cells (iPSCs). Eventually, these models may help uncover potential targets for therapeutic intervention on both stromal and immunological basis.

## Clinical Implementation of Immune Suppression in PAH

Over the last decades great progress was made on the development of novel therapies in PAH. Examples of these are the classes of endothelin-1 receptor antagonists (ERAs), phosphodiesterase-5 inhibitors (PDE5i), and the prostacyclins, which can now also be administered orally, or prostacyclin receptor agonists like selexipag. Interestingly, these therapies mainly focus on vasodilation of the pulmonary vasculature to lower intrapulmonary pressure, but despite their efficacy these are not true curative therapeutic options, and life expectancy and quality of life for PAH patients is still relatively low. It is also intriguing that it is unknown and not well studied how these therapies impact the vascular remodeling. The same holds true for the effects of these compounds on the immune phenomena that were involved in the disease process. As we extensively elucidated the role of the immune system in vascular remodeling in PAH throughout this review, we anticipate that the immune system may be a promising therapeutic target. This could be important, as immunological mechanisms contribute to irreversible vascular remodeling and its progression should be halted as early as possible.

Nearly all the aforementioned preclinical studies suggest a major role for IL-6 in vascular remodeling in PAH. This makes IL-6 an interesting potential therapeutic target. The use of tocilizumab (anti-IL-6-receptor blocker) has been investigated for itsefficacy in PAH, in a randomized phase 2 clinical trial (www.clinicaltrials.gov NCT02676947) (69, 70). Six months of treatment with tocilizumab, in this selection of patients, showed no significant improvement of PVR. However, this study had some major limitations. Simpson et al. showed that IL-6 levels vary in different subtypes of PAH, and other studies already showed that IL-6 in patients with for instance COPD are very low, compared to IPAH or CTD-aPAH (61, 62). Tocilizumab may therefore not be the drug of choice in patients without supportive evidence for underlying IL-6 mediated pathology. This randomized controlled trial (RCT) included world health organization (WHO) type 1 PAH patients, though CTD-aPAH patients with underlying disease like mixed connective tissue disease (MCTD), SLE and, especially, rheumatoid arthritis (RA) were excluded (69, 70). Toshner et al. argued that PAH in these patients may be rare and that they, in many cases, already used immunosuppression.

Studies on the use of immunosuppressive agents in PAH associated with SLE/MCTD exist but are limited in number (71–74). In the paradigm disease for PAH, namely SSc-aPAH and in rare diseases like the anti-synthetase syndrome associated with PAH, only a few case reports are published (75, 76). This is remarkable, as immune activity and autoimmunity is clearly present in these patients. One reason will be that in the majority of these patients the development of PAH is a late manifestation, and treatment is considered by many to be outside the window of opportunity. It is therefore important to identify as early as possible the still reversible immunological phase in the disease process. It is also still a challenge to identify and select the optimal target of immune suppression for the individual patient, depending on the dominant underlying mechanism. Selecting a broad range of patients with different underlying pathologies, be it immune mediated or not, may hamper the final results when studying the effects of, for instance, tocilizumab. Tocilizumab has already been investigated as a monotherapy in RA with favorable results (77). It therefore seems less logical to exclude patients from a trial in which a common pathogenic cytokine is targeted. In SSc, the paradigm vasculopathic disease for PAH, IL-6 serum levels were also increased when compared to healthy controls and correlated with disease severity (78). Nevertheless, due to the exclusion criteria, the major etiology of CTD-aPAH in the study of Toshner et al. was SSc (70). This study showed that more patients with CTD-aPAH had a >15% reduction in PVR after 6 months of tocilizumab treatment, in comparison to IPAH/HPAH patients. Therefore, tocilizumab could still be an interesting therapeutic option in SSc-aPAH, and should be specifically investigated in the future.

The effects of immunosuppression in PAH could be less easy to measure than the direct effect of vasodilators, but it will most likely contribute to the halting progression of the pathogenic vascular remodeling. Outcome measurements should focus on survival, maintenance of functional class (NYHA), diffusion capacity, or 6 min walking distance in addition to pulmonary vascular resistance or mean pulmonary arterial pressure (mPAP). Novel therapeutic options like sotatercept are currently under investigation and show promising results in decreasing PVR, by blocking activins and restoring anti-proliferative balance of the BMPR2 (bone morphogenetic protein receptor 2) pathway (79), suggesting reversibility in vascular remodeling. Research on these new agents should also focus on the effects of immune cells that contribute to vascular remodeling. Treatment for PAH in the future will most likely consist of a combination of medication that affects different mechanisms in the development of PAH. Vasodilative agents will quickly reduce right ventricular systolic pressure (RVSP), while immune modulation will halt or slow disease progression and vascular remodeling. In combination with agents that stimulate reversibility of the remodeled vessels a better outcome for the patient is anticipated.

## Discussion

Due to the elaborate dynamic interplay between different aspects of vascular stromal cells, chemokines, cytokines, complement proteins and immune cells of both the innate and adaptive immune system, it is hard to quickly summarize the underlying process in PAH. This review established a broad view of the many roles of the immune system in PAH, especially focusing on vascular remodeling. Endothelial injury may act as a trigger, inducing cytokine and chemokine responses, producing vasoconstrictive agents like endothelin-1 and decreased eNOS, and possibly activating the complement cascade leading to more inflammation. Damaged endothelial cells become dysfunctional and directly stimulate vascular stromal cells such as PASMCs to proliferate. Pericytes that regulate homeostasis of proliferation in vessels become dysfunctional, and differentiate into PASMCs themselves, losing their regulating function. Besides, local cells like fibroblasts and mast-cells are activated as well. These cells produce cytokines like IL-6 that have direct effects on vascular stromal proliferation and cell wall thickening, but also activate other immune cells like B-cells, stimulating more antibody-production and perivascular inflammation. Besides, macrophages polarize into a pro-fibrotic vascular repair type mediated by IL-6, that act in an overshooting manner excreting matrix protein, inducing vascular fibrosis and even stimulate proliferation of PASMCs. Fibroblasts may also differentiate into PASMC types and migrate into the medial layer inducing medial thickness, and neointima formation.

## Conclusion

By definition, PAH is a progressive pan-vasculopathic cardiopulmonary disease caused by overshooting immunological and stromal “repair” mechanisms, eventually leading to decreased quality of life of patients, with risk of cardiac failure and premature death. PAH is known for its broad etiology and pathogenesis. However, in the clinical approach of patients with PAH the identification of underlying active immune involvement is crucial, being one of the driving factors of disease progression. Immune activation, as evidenced by the presence of circulating autoantibodies, composition and phenotype of lymphocyte subsets, and other soluble biomarkers, should be explicitly investigated in clinical patients. Future studies should focus on the selective use of immunosuppressant's and immunomodulatory agents in PAH to stop disease progression. It would also be of interest to study the immunomodulatory effect of the present vasoactive agents as well novel agents like the activin antagonist sotatercept. Integration of models in which the patient's immune cells, serum and vascular stromal cells meet, could be essential for a better understanding of the process of a dynamic disease like PAH. Collaboration between clinicians and fundamental researchers is therefore key in the progress of treating this multi-factorial and devastating disease.

## Author Contributions

RT wrote the concept of the review which was adapted to the final version by the other authors. RY prepared the figures. The final version was approved by all authors. All authors contributed to the concept of the review.

## Conflict of Interest

The authors declare that the research was conducted in the absence of any commercial or financial relationships that could be construed as a potential conflict of interest.

## Publisher's Note

All claims expressed in this article are solely those of the authors and do not necessarily represent those of their affiliated organizations, or those of the publisher, the editors and the reviewers. Any product that may be evaluated in this article, or claim that may be made by its manufacturer, is not guaranteed or endorsed by the publisher.

## Abbreviations

5-HT: 5 hydroxy-tryptophane
AECAs: Anti-endothelial cell antibodies
AngII: Angiotensin 2
Anti-dsDNA: Anti-double stranded DNA
APS: Anti-phospholipid syndrome
BMPR2: Bone morphogenetic protein receptor 2
C3d: Complement degradation product d
CCL2: C-C motif ligand 2
CCL5: C-C motif ligand 5
CD: Cluster of differentiation
Cfb: Complement factor b
COPD-aPAH: Chronic obstructive pulmonary disease associated pulmonary arterial hypertension
CTD-aPAH: Connective tissue disease associated pulmonary arterial hypertension
CTEPH: Chronic thromboembolic pulmonary hypertension
CXCL12: C-X-C motif ligand 12
ECM: Extracellular matrix
eNOS: Endothelial nitric oxide synthase
ERAs: Endothelin receptor antagonists
ET-1: Endothelin 1
FGF-2: Fibroblast growth factor 2
GM-CSF: Granulocyte macrophage colony stimulating factor
HPAH: Hereditary pulmonary arterial hypertension
HPH: Hypoxic pulmonary hypertension
HUVEC: Human umbilical vein endothelial cell
ICAM-1: Intercellular adhesion molecule-1
IL-1: Interleukin 1
IL-13: Interleukin-13
IL-17: Interleukin-17
IL-21: Interleukin-21
IL-4: Interleukin-4
IL-6: Interleukin 6
IPAH: Idiopathic pulmonary arterial hypertension
iPSC: Inducible pluripotent stem cell
LTB-4: Leukotriene B4
MCP-1: Monocyte chemoattractant protein 1
MCT: monocrotaline
MMP-2: Matrix metallo-proteinase 2
mPAP: Mean pulmonary arterial pressure
NO: Nitric Oxide
NYHA: New York heart association
PAH: Pulmonary arterial hypertension
PASMC: Pulmonary artery smooth muscle cell
PAWP: Pulmonary arterial wedge pressure
pDCs: Plasmacytoid dendritic cells
PDE5i: phosphodiesterase-5 inhibitors
PDGF-B: platelet derived growth factor-B
PDK4: Pyruvate dehydrogenase kinase 4
PGI2: Prostaglandin I2
PH: Pulmonary hypertension
PHFib: Pulmonary hypertension fibroblast
PVOD: Pulmonary venous occlusive disease
PVR: Pulmonary vascular resistance
RANTES: Regulated upon activation normal T cell expressed and presumably secreted
RCT: Randomized controlled trial
RHC: Right heart catheterization
SDF-1: Stromal cell derived factor 1
SiMFis: Singular millimetric fibrotic lesions
SLE: Systemic lupus erythematosus
SSc: Systemic sclerosis
STAT3: Signal Transducer And Activator Of Transcription 3
STAT6: Signal Transducer And Activator Of Transcription 6
TCR-γδ: T-cell receptor γδ
Th17: T-helper 17
VATS: Video assisted thoracic surgery
VCAM-1: Vascular cellular adhesion molecule 1.
